# Characterization of the Staphylococcal Cassette Chromosome Composite Island of *Staphylococcus haemolyticus* SH32, a Methicillin-Resistant Clinical Isolate from China

**DOI:** 10.1371/journal.pone.0087346

**Published:** 2014-01-23

**Authors:** Dongliang Yu, Borui Pi, Yan Chen, Yanfei Wang, Zhi Ruan, Michael Otto, Yunsong Yu

**Affiliations:** 1 Institute of Developmental and Regenerative Biology, Hangzhou Normal University, Hangzhou, China; 2 Department of Infectious Diseases, Sir Run Run Shaw Hospital, College of Medicine, Zhejiang University, Hangzhou, China; 3 National Institute of Allergy and Infectious Diseases, The National Institutes of Health, Bethesda, Maryland, United States of America; Institut National de la Recherche Agronomique, France

## Abstract

Staphylococcal cassette chromosome (SCC) elements contribute considerably to virulence and resistance to antibiotic agents in staphylococci. SCC elements in coagulase-negative staphylococci (CoNS) are highly diverse and there is evidence suggesting that they serve as a reservoir for antibiotic resistance genes in methicillin-resistant *Staphylococcus aureus* (MRSA). However, only a small number of SCC elements have been characterized in CoNS and their exact roles in the emergence and evolution of MRSA remain to be demonstrated. Here, we determined the structure of an SCC composite island (CI*_SH32_*) found in the clinical *Staphylococcus haemolyticus* isolate SH32 by whole-genome DNA sequencing. CI*_SH32_* was 48 kb in length and mainly composed of two imperfect SCC elements, namely (i) a *Ψ*SCC*mec*(*SH32*) part containing a class C1 *mec* gene complex but lacking *ccr* genes and (ii) a SCC*_SH32_* part with a *ccrA5B3* gene complex but lacking *mec* genes. In addition, CI*_SH32_* contained a type III restriction-modification system and several resistance loci, for example genes conferring resistance to cadmium and arsenic. *Ψ*SCC*mec*(*SH32*) is almost entirely identical to a pseudo SCC*mec* element found in *S. haemolyticus* WCH1 and shares pronounced sequence similarity to a *Ψ*SCC*mec* element of *S. haemolyticus* JCSC1435. However, staphylococci other than *S. haemolyticus*, including *S. aureus* and *S. epidermidis*, contain homologs of SCC*_SH32_* that are more similar to SCC*_SH32_* than those elements found in *S. haemolyticus*, suggesting that CI*_SH32_* of *S. haemolyticus* SH32 was assembled in recent evolutionary events. Moreover, the composite structure of CI*_SH32_* indicates that the detection of class C1 *mec* and *ccrA5B3* gene complexes in *S. haemolyticus* does not always indicate the existence of a UT9-type SCC*mec* element, which has remained questionable.

## Introduction

The emergence and spread of methicillin-resistant *Staphylococcus aureus* (MRSA) has become a worldwide problem, which is in part due to the extensive repertoire of virulence factors present in many MRSA strains and the fact that some isolates also contain genes providing resistance to a wide array of other antibiotic agents [1]. Virulence and antibiotic resistance genes are mostly encoded by mobile genetic elements (MGEs), including plasmids, transposons and staphylococcal cassette chromosome (SCC) elements [2]. SCC*mec* elements represent a particular concern, because they harbor *mec* genes (*mecA/mecC*) providing resistance to methicillin and almost all other beta-lactam antibiotics.

In recent years, an extensive genetic diversity of SCC*mec* elements has been revealed in *S. aureus* and other staphylococci, by PCR assays, DNA microarrays, or genome sequencing [3]–[7]. These studies also showed that coagulase-negative staphylococci (CoNS) harbor more diverse SCC*mec* elements than *S. aureus* and are a potential reservoir for the transfer of SCC*mec* elements to *S. aureus* [8]. However, the exact role of SCC*mec* elements of CoNS in the emergence and evolution of MRSA is largely unknown and requires characterization of more SCC/SCC*mec* elements. In the present work, we identified an SCC composite island (CI) in the clinical *S. haemolyticus* isolate SH32 and analyzed its structure in comparison to related SCC elements.

## Materials and Methods

### Ethics statement, strain and DNA preparation

*S. haemolyticus* SH32 was isolated in 2003 from the blood of an inpatient at First Affiliated Hospital, College of Medicine, Zhejiang University. We obtained an exempt status from the Institutional Review Board of the First Affiliated Hospital, College of Medicine, Zhejiang University to use this strain to perform all experiments in this study. Antibiotic susceptibility of the strain was described previously [9]. Genomic DNA was prepared with a QIAamp DNA mini kit (Qiagen) according to the manufacturer's instructions.

### Sequencing and gap closure

Both pair-end and mate-pair (3-kb insertion) libraries were constructed and sequenced using a HiSeq 2000 platform (Illumina, USA) in the Chinese National Human Genome Center, Shanghai. The acquired 2×100 bp reads were assembled by velvet software [10]. Identification of contigs representing parts of SCC elements was performed using BLAST tools [11], using mapping marker genes such as *orfX*, *mecA* and the *ccr* gene complex. Gap closure was then performed using standard PCR and PCR product sequencing using a 3730XL instrument (Applied Biosystems, USA).

### Annotation and comparative analysis

Gene prediction in *S. haemolyticus* SH32 was performed using GeneMark-hmm (V2.08) [12]. The nucleotide acid sequence of predicted genes and their deduced amino acid sequences were subsequently compared against the non-redundant protein database provided by NCBI (www.nlm.nih.gov) using BLAST tools [11].

The comparison of different SCC elements was performed using BLASTN and displayed by in-house developed perl scripts. The sequences used in the comparative analysis were retrieved from the NCBI website.

### Detection of mecA and ccrA5B3

A specific primer pair (forward: GAACCGCAGGTCTCTTCAGATCTAC; reverse: CACCTTGTCCGTAACCTGAATC) was used to determine the class C1 *mec* gene complex using standard PCR. Long-distance PCR was performed to detect *ccrA5B3* (forward: CGCGCTATTATCACGAATCC; reverse: GCGTGATTAAGTGCGTTAGC). The PCR products were subsequently sequenced using an ABI 3730 sequence analyzer (Applied Biosystems).

## Results and Discussion

### Assembly and annotation

**Figure 1:**
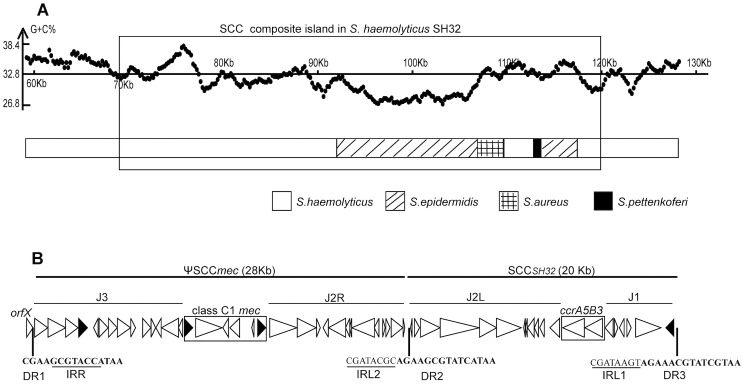
General features of the SCC composite island in *Staphylococcus haemolyticus* SH32. (A) Distribution of the best-hit homologs of genes contained in the SH32 SCC composite island in staphylococci. The G+C content of the SCC region (from 70 to 120 kb) was calculated with a window size of 200 bp. 32.8% is the average G+C content of the whole chromosome. (B) Gene structure of the SH32 SCC composite island. Predicted genes in SCC elements are represented by triangles, while their direction indicates the encoding strands. Black triangles denote IS*431* genes. Abbreviations: DR, direct repeat sequence; IR, inverted repeat sequence.

Genome sequencing of *S. haemolyticus* SH32 generated 339 contigs. Five of them harbored *orfX*, *mecA* and *ccr* genes or fragments, indicating that they contain fragments of an SCC*mec* element. The gaps between these contigs were then closed by standard PCR and PCR product sequencing, which yielded one contig with a length of 197,413 bp and an average G+C content of 33.3% (accession number: KF006347). Among a total of 201 genes that were identified in this contig, most had their best-hit homologs in *S. haemolyticus* JCSC1435. Almost half of the genes in the identified SCC composite island (hereafter referred to as CI*_SH32_*) were more similar to their counterparts in *S. epidermidis*, *S. aureus* and other staphylococci than those reported from *S. haemolyticus* (Figure 1A). Partially, this may be due to the limited information available about SCC/SCC*mec* elements in *S. haemolyticus*, but it may also suggest events of horizontal transfer of those SCC*mec* elements between the respective staphylococcal species.

### General features of the SCC composite island in SH32

The CI*_SH32_* region, which is characterized by the bracketing 15-bp sequences found in integration site sequences containing directed (DR) and inverted repeats (IR) (Figure 1B), is about 48 kb in length and comprises 54 predicted genes (SHP0065-118) (Table 1). The G+C content of CI*_SH32_* is 31.6%, slightly lower than that of the whole chromosome of *S. haemolyticus* (∼32.8%), which is possibly due to an enrichment of genes originating from horizontal gene transfer in SCC elements. In contrast to the composite islands found in *S. epidermidis* ATCC 12228 and *S. haemolyticus* JCSC1435, for which eight and six copies, respectively, of integration site sequences (ISS) were found and which are composed of multiple SCC elements (or remnants) [13], [14], only two copies of ISS were found in CI*_SH32_* in addition to that present in *orfX*. This suggests that less complex integration events occurred in the SCC element in *S. haemolyticus* SH32. According to the nomenclature of IWG-SCC [15], the structure of CI*_SH32_* was described as *orfX*-ΨSCC*mec*(*SH32*)-SCC*_SH32_*, *i.e.* a pseudo SCC*mec* element that contains a *mec* gene complex but lacks *ccr* recombinases [hereafter referred to as ΨSCC*mec*(*SH32*)], and an SCC element that harbors *ccr* but lacks *mec* genes (hereafter referred to as SCC*_SH32_*).

**Table 1:** Gene content of the SCC*mec* element in *Staphylococcus haemolyticus* SH32.

Locus	Position^1^	Regions	Characteristic genes	Description
SHP0065	70295–70774		*orfX*	rRNA large subunit methyltransferase
SHP0066	70903–71889	J3		ADP-ribosylglycohydrolase
SHP0067	71908–73242			Permease
SHP0068	73239–74177			truncated ribokinase
SHP0069	74215–74889		IS*431*	transposase for IS431*mec*
SHP0070	75693–75346			transcriptional regulator
SHP0071	75771–76454			ThiJ/PfpI family protein
SHP0072	76476–77141			NAD dependent epimerase/dehydratase
SHP0073	77145–78149			Oxidoreductase
SHP0074	78158–78523			hypothetical protein
SHP0075	79059–79676		*cadD*	cadmium binding protein CadD
SHP0076	79695–80036		*cadX*	cadmium resistant accessory protein
SHP0077	80457–80056		*arsC*	arsenate reductase
SHP0078	81764–80475		*arsB*	arsenical pump membrane protein
SHP0079	82081–81764		*arsR*	arsenical resistance operon repressor
SHP0080	82211–82885	*mec* gene complex	IS*431*	transposase for IS-like element
SHP0081	83061–85067		*mecA*	penicillin-binding protein 2′
SHP0082	85541–85113			uncharacterized protein ydem
SHP0083	86381–85638			glycerophosphoryl diester phosphodiesterase
SHP0084	87465–87298			3-hydroxy-3-methylglutaryl CoA synthase
SHP0085	87723–88397		IS*431*	transposase for IS-like element
SHP0086	88660–90720	J2R	*copA*	P-type ATPase copper (Cu2+) transporter
SHP0087	90735–92168			multicopper oxidase mco
SHP0088	92188–92478			putative lipoprotein
SHP0089	93079–92684		*arsC*	arsenate reductase
SHP0090	94390–93098		*arsB*	arsenite-antimonite efflux pump
SHP0091	94704–94390		*arsR*	arsenic resistance operon repressor
SHP0092	94844–94701			hypothetical protein
SHP0093	96574–94844		*arsA*	arsenite-activated ATPase
SHP0094	96902–96555		*arsD*	arsenical resistance operon trans-acting
				repressor
SHP0095	97185–97379			hypothetical protein
SHP0096	97425–97745			ArsR family transcriptional regulator
SHP0097	97833–98717			putative permease
SHP0098	98731–98859			hypothetical protein
SHP0099	99411–99295	J2L		hypothetical protein
SHP0100	99585–99977			type III R-M system enzyme, M subunit
SHP0101	100048–101532			type III R-M system protein
SHP0102	101534–104503			type III R-M, res subunit
SHP0103	104510–105802			hypothetical protein
SHP0104	105786–107660			type III R-M, res subunit
SHP0105	107961–107842			hypothetical protein
SHP0106	108112–107981			hypothetical protein
SHP0107	108630–108127			hypothetical protein
SHP0108	108959–108648			hypothetical protein
SHP0109	109396–109046			hypothetical protein
SHP0110	110695–109961			cyclopentanol dehydrogenase
SHP0111	112423–110777	*ccr* gene complex	*ccrB*	cassette chromosome recombinase B
SHP0112	113793–112459		*ccrA*	cassette chromosome recombinase A
SHP0113	114595–113981	J1	*cch*	cassette chromosome helicase
SHP0114	114682–114957		*ΨccrA*	cassette chromosome recombinase A
SHP0115	115428–115069			zinc/iron permease
SHP0116	115635–115961			HTH-type transcriptional repressor CzrA
SHP0117	116291–118216			cadmium-translocating P-type ATPase
SHP0118	119212–118538		IS*431*	transposase for IS*431mec*

1 gene position in the contig (accession number: KF006347).

### *Ψ*SCC*mec*(*SH32*) is conserved in *S. haemolyticus*

*Ψ*SCC*mec*(*SH32*) is about 28 kb in length (as determined by the size of the DNA region between DR1 and DR2), and contains three regions, a *mec* gene complex, a J3 region and a J2R region. The *mec* gene complex belongs to the C1 class with the common structure *IS431*-*mecA*-*ΔmecR1*-*IS431*, with two copies of IS431 arranged in the same direction. J2R, the region between the *mec* gene complex and DR2, is about 10 kb in length and encodes two heavy metal resistance genes/gene clusters, *copA* and *arsCBRAD*, conferring resistance to copper and arsenic, respectively. Interestingly, the J3 region (extending from IRR to the *mec* gene complex) harbors another cluster of arsenic resistance genes (*arsCBR*). The corresponding copies of the *arsC*, *arsB* and *arsR* genes in J2R and J3 share 84%, 86% and 63% amino acid identity, respectively. However, homologs of *arsCBR* and *arsCBRAD* with higher similarity were found in *S. epidermidis* and *S. haemolyticus* isolates, indicating that these two *ars* clusters in *S. haemolyticus* SH32 were not generated by duplication and divergence, but are derived from different ancestors. Finally, the J3 region contains additional cadmium resistance genes, *cadD* and *cadX*.

**Figure 2:**
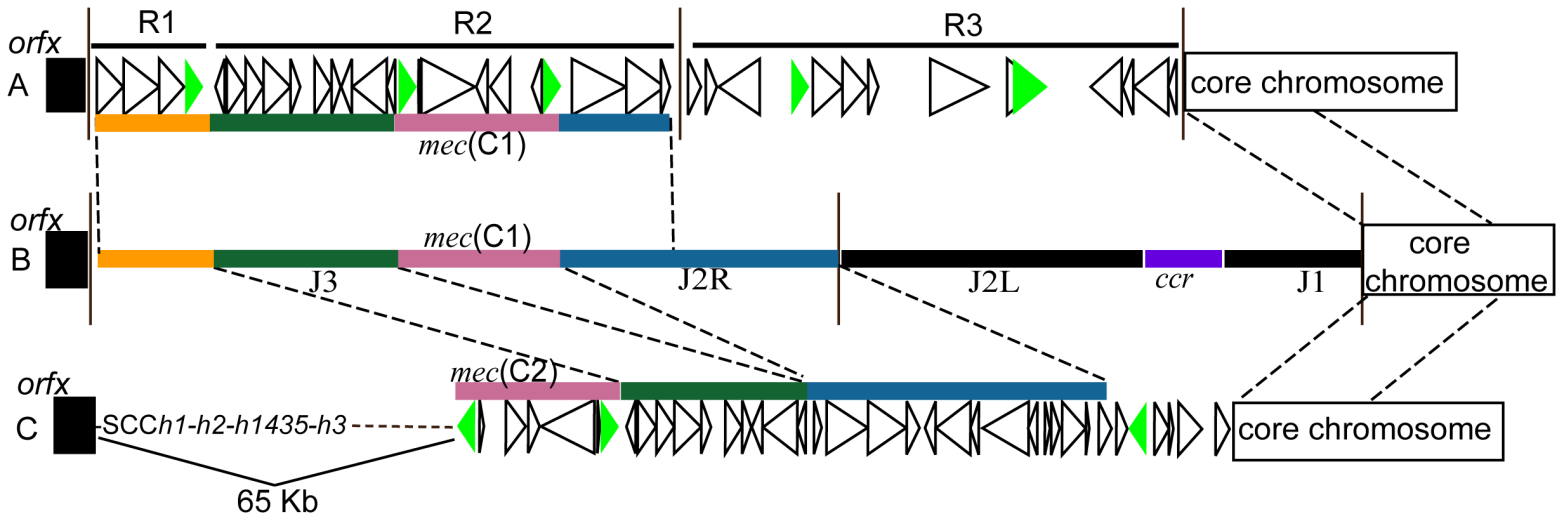
Comparison of SCC*mec* elements in three *Staphylococcus haemolyticus* isolates. A: WCH1; B: SH32; C: JCSC 1435. *mec*(C1/C2): class C1/C2 *mec* complex. Rectangles with the same colors denote homologous regions. Dashed lines indicate their ends. Vertical solid lines indicate the site of integration site sequences. Green triangles denote insertion sequences.

*Ψ*SCC*mec*(*SH32*) is largely identical to *Ψ*SCC*_WCH1_* (Figure 2). However, *Ψ*SCC*_WCH1_* harbors an accessory region downstream of DR2 (ca. 17 kb, designated R3), which contains two IS elements, IS*431* and IS*Sha1*, as well as several genes encoding putative bacterial virulence factors, such as the proline permease PutP and the ion transporter FeoB [16], [17]. In contrast, *Ψ*SCC*mec*(_SH32_) contains an accessory 6-kb insertion between the *lip* gene (SHP0088, encoding a lipoprotein) and DR1-2 (designated J2R-6k), which mainly encodes the *ars* gene cluster and is conserved in *S. haemolyticus* JCSC1435 (SH0089-98). Given the different positions of the R3 regions in *Ψ*SCC*_WCH1_* (*lip*-ISS-insertion) and the J2R-6k in *Ψ*SCC*mec*(*SH32*) (*lip*-insertion-ISS), it is apparent that they were acquired by independent events. Zong *et al*. proposed that the R3 region resulted from homologous recombination, as no DR could be identified in the junction region [18]. However, considering that the J2R-6k region is well conserved in the isolates SH32 and JCSC 1435, this is most probably a common feature of a hypothetical ancestral *S. haemolyticus* clone that was lost in the isolate WCH1.

### Comparative analysis of SCC*_SH32_*

SCC*_SH32_*, bracketed by DR2 and DR3, is about 20 kb in length and divided into three regions, namely a *ccr* gene complex and the J2L and J1 regions. The recombinase genes in SCC*_SH32_* are of the types *ccrA5* and *ccrB3* and are 1,335 bp and 1,647 bp in length, respectively. They are identical to those of *S. haemolyticus* H9, which were originally reported as *ccrB*_SHP_ and *ccrA*_SHP_ [9]. A *ccrA5B3* complex has also been identified in another CoNS, *S. cohnii* WC28 (accession number GU370073), and the coagulase-positive *S. pseudintermedius* KM241 (accession number AM904731, originally reported as *ccrA5B5*) [19], [20]. The *ccrA5* gene of SCC*_SH32_* shares 89.7% and 91.5% nucleotide identity, and the *ccrB3* gene 87.7% and 86.4%, with the corresponding genes of the SCC*mec* elements of *S. cohnii* WC28 and *S. pseudintermedius* KM241, respectively. Using a similarity search in the non-redundant NCBI nucleotide database, we also found highly similar (>99% identity) *ccr* gene complexes in four *S. aureus* isolates (accession numbers: HF569102, HF569097, HF569093 and GU066221).

J1, the region extending from the *ccr* gene complex to IRL1, is about 5.5 kb in length and contains six genes (SHP0113-118), including a truncated *ccrA* gene and genes encoding a cassette chromosome helicase and two ion permeases. Comparative analysis revealed that the J1 region is almost identical to those found in SCC*pbp4* of *S. epidermidis* ATCC 12228 (accession number BK001539) and SCC elements in *S. aureus* isolates M1 (accession number HM030720, type IV) and BK20781 (accession number FJ670542, type VIII). The J2L region in SCC*_SH32_*, *i.e.* the region between the *ccr* gene complex and DR2, is about 10 kb in length. In contrast to type I restriction-modification (R-M) systems, such as *hsdR*, *hsdS* and *hsdM*, identified in most known SCC*mec* elements, a type III R-M system was found in SCC*_SH32_* J2L. Interestingly, this R-M system is almost identical to an R-M system found in *S. epidermidis* (accession number NZ_AKGM01000027). In addition, we identified several hypothetical proteins in J2L, for example SHP0106-109, homologs of which are found in *S. haemolyticus* JCSC 1435 and isolates from other staphylococcal species like *S. epidermidis* and *S. aureus*. SHP0107 and SHP0108 are conserved in both species with an identity value of >92%, but SHP0106 and SHP0109 show higher similarity (98% and 97% identity values, respectively) to their counterparts in *S. aureus* or *S. epidermidis* than *S. haemolyticus* JCSC1435 (86% and 55%, respectively).

The *ccrA5B3* gene complex has been identified in several staphylococcal species, both coagulase-negative and coagulase-positive staphylococci, with varied identity, revealing that it was exchanged between staphylococcal species during evolution. Remarkably, although *ccr* genes encoded by SCC*_SH32_* are not found in *S. epidermidis*, several *S. epidermidis* isolates contain regions that are highly homologous to the two junction regions in SCC*_SH32_*, J1 and J2L, which include the type III R-M system. R-M systems are responsible for limiting the uptake of foreign DNA in bacteria; and both types I and III R-M systems have been identified as major barriers to lateral gene transfer in *S. aureus* [21]–[23]. Therefore, the apparent frequent gene transfer between *S. haemolyticus* and *S. epidermidis* in SCC regions may be ascribed to the fact that they have similar R-M systems.

### Detection of *ccrA5B3* in methicillin-resistant *S. haemolyticus*

We collected a total of 88 methicillin-resistant *S. haemolyticus* (MRSH) isolates in previous work [9], including 42 isolates that are positive for the *arcA* gene of the arginine catabolic mobile element (ACME) [24]_ENREF_10. By using PCR amplification and dot blotting, class C1 *mec* gene complexes and the *ccrA5B3* gene were detected in eight of these 42 isolates, including the isolate SH32 [9]. In this work, we investigated the other 46 ACME-*arcA* negative isolates and found that another two isolates harbored *ccrA5B3* gene complexes. Interestingly, both of them also contained class C1 *mec* gene complexes, suggesting that these isolates diverged after the assembly of class C1 *mec* and *ccrA5B3* gene complexes.

### Concluding remarks

More detailed knowledge of CoNS SCC*mec* elements is of special importance, as they are believed to serve as reservoirs for SCC*mec* elements transferred to *S. aureus* [25]. In this study we characterized the entire structure of a SCC composite island in *S. haemolyticus*, which we found is composed of two SCC remnants. Results from a previous study suggested the presence of a UT9-type SCC*mec* element in *S. haemolyticus* that encodes both a class C1 *mec* and a *ccrA5B3* gene complex [6]. Notably, we here found that these two gene complexes are present in two separate SCC remnants, and are not assembled in one SCC*mec* element. Although the class C1 *mec* complex and *ccrA5B3* genes have been identified in several other *S. haemolyticus* isolates, our results indicate that confirming the existence of a UT9 SCC*mec* element solely by PCR assays is problematic, necessitating the analysis of further genome sequences.
